# Bioactive Natural Products 2018

**DOI:** 10.1155/2018/5063437

**Published:** 2018-05-27

**Authors:** Yiannis Kourkoutas, Nikos Chorianopoulos, Veronica Lazar, Pierluigi Di Ciccio

**Affiliations:** ^1^Laboratory of Applied Microbiology and Biotechnology, Department of Molecular Biology and Genetics, Democritus University of Thrace, 68100 Alexandroupolis, Greece; ^2^Institute of Technology of Agricultural Products, Greek Agricultural Organization Demeter, 15310 Athens, Greece; ^3^Laboratory of Microbiology-Immunology, Faculty of Biology, Department of Botany-Microbiology, University of Bucharest, Romania; ^4^Food and Drug Department, University of Parma, Italy

The global phenomenon of antibioresistance, resistance genes pool (clinical and environmental reservoirs), and environmental pollution, especially by xenobiotics, including antibiotics which are considered water micropollutants, are acknowledged as some of the most important problems the world is facing today [1]. Thus, the necessity of antimicrobial agents that are new, efficient, non-toxic, and with no selective pressure activity is obvious.

Despite the huge scientific progress in vaccination and chemotherapy, infectious diseases remain a serious health issue. Under the selective pressure of therapeutical antibiotics, used excessively during the last decades, some bacterial species/strains harboring resistance genes (pre-existent to antibiotherapy) were selected and disseminated, developing other mechanisms of resistance. As a consequence, infectious diseases remain among the leading causes of morbidity worldwide and a top priority for the public health. However, little progress has been made in the development of new antimicrobial drugs. Moreover, the wide use of antibiotics has evolutionary and ecological effects, leading to the recruitment of more genes into the* resistome* and* mobilome*, with adverse consequences for human welfare and environment [2, 3]. There are also a lot of biofilm-associated infections and the biofilm embedded cells show a different form of resistance, called now tolerance. Biofilms cause great medical concerns, as they may be developed on medical devices, tissues, and organs (normal or damaged), but also industrial problems, since they could be formed on any device and industrial equipment. Microorganisms attached to a substratum and organized in biofilms exhibit a high tolerance to the current antibiotics, antiseptics, and biocides, as well as to the host defense mechanisms. Moreover, the resistance and virulence genes are easily achieved between biofilm's embedded cells by horizontal transfer, due to their proximity [4].

In the industrial environment, current anti-fouling agents are also far from being efficient. Hence, the adherent microorganisms on surfaces produce great economical losses caused by the uncontrolled development of biofilms on pharmaceutical or food industrial equipment. Therefore, numerous industrial technologies have to make a difficult choice: either to utilize a high amount of an efficient anti-fouling agent with the risk of developing side effects and impurifications on the final product or not to be able to control the microbial contamination and biofilm development within the technological processes. In these conditions, new, safe for health (without cytotoxicity), and eco-friendly biocides are necessary, because the consumers are currently informed, show great interest, and demand healthy food. In the coming years, it is estimated that the EU regulations will be changed and certain biocides will be banned, due to their biohazard effects.

Considering the high frequency of genetic antibioresistance in the most common pathogens, the huge public health burden of severe biofilm-associated infections (60–80% of all infections), and the great economical loses caused by the uncontrolled development of biofilms on industrial equipment, alternative strategies are urgently needed to efficiently control their formation and their negative effects. Thus, the researchers are in a continuous quest for new antibacterial agents for resistant/multiresistant strains, able to penetrate the biofilms and with activity on adherent cells. Innovative approaches include the following: (1) the development of prophylactic antimicrobial peptides, able to interfere with the intercellular communication by quorum-sensing (QS) mechanism, involved in regulation of a series of genes, including virulence genes; such QS inhibitors (QSIs) belong to the antipathogenic strategies [5]; (2) enzymes able to degrade biofilm's matrix (dispersins) or the signal molecules (quenching enzymes). To date, none of the envisaged antibiofilm solutions has an absolute outcome, but only their combinations seem to be effective.

The use and abuse of antibiotics, especially those with large spectrum of activity, are the cause of the frequent condition of disbiosis or alteration of the intestinal microbiota's interspecific equilibrium. Such conditions are leading to opportunistic infections, metabolic disturbances, increased intestinal permeability, and chronic inflammation. Evidence obtained by animal models and clinical studies confirm the association of an altered gut microbiota with all corollary consequences, such as metabolic diseases from obesity to type-2 diabetes, tooth decay, cardiovascular diseases, and cancer [6].

All these recently high increased problems have catalyzed the research efforts to find new ways to fight against pathogens, with no side effects on the host and its normal microbiota, but also on the environment. A lot of studies are now focused on the investigation of bioactive natural products (BIONPs), mainly obtained from plants with a very wide range of biological activities: antimicrobial, anti-inflammatory, antioxidant, immunomodulatory, antidepressant, antihyperglycaemic (amylase activity), antihypertensive, anticarcinogenic, etc. These BIONPs are used as plant extracts or fractions, coupled or not with carriers (nanoparticles). Medicinal plants have now to be investigated at molecular level, in order to identify the mechanisms of action, efficiency, and lack of cytotoxicity, since their use has to be scientifically based, in definite amounts and for a specific target, in comparison with the allopathic drugs. The potential synergistic activity with antibiotics should be also explored [7, 8].

Thus, plants are an important source of BIONPs; all plants have immune defense mechanisms mediated by anti-infectious phytocompounds, such as phytoanticipins and phytoalexins and the more recently described QSIs. The QSIs exhibit, when used even in subinhibitory concentrations, an indirect antimicrobial effect, manifested by inhibiting the bacterial intercellular communication by QS mechanism and coordinated expression of virulence genes depending on cellular density. The use of QSIs could represent an efficient and intelligent strategy to control resistance/tolerance, virulence, and colonization/biofilm formation, without selective pressure and other side effects [5, 9–13].

However, the use of BIONPs has some limitations, due to their low availability and stability, high volatility, and a great diffusion ability that do not recommend their implementation in the current medical practice. These features lead to the necessity of developing vectorization and delivery agents for improving their efficiency and also optimized assay methods adapted for their specific properties. However, the research efforts are fully justified by their great potential.

*Yiannis Kourkoutas* *Nikos Chorianopoulos* *Veronica Lazar* *Pierluigi Di Ciccio*
